# Cortical Bone Derived Stem Cells Modulate Cardiac Fibroblast Response via miR-18a in the Heart After Injury

**DOI:** 10.3389/fcell.2020.00494

**Published:** 2020-06-23

**Authors:** Lindsay Kraus, Lena Ma, Yijun Yang, Faustina Nguyen, Robert C. Hoy, Tomoko Okuno, Mohsin Khan, Sadia Mohsin

**Affiliations:** ^1^Independence Blue Cross Cardiovascular Research Center, Lewis Katz School of Medicine, Temple University, Philadelphia, PA, United States; ^2^Center for Metabolic Disease, Lewis Katz School of Medicine, Temple University, Philadelphia, PA, United States

**Keywords:** stem cells, cardiac fibrosis, myocardial infarction, fibroblasts, miR-18a

## Abstract

The adult heart following injury such as a myocardial infarction forms a fibrotic scar associated with transformation of resident cardiac fibroblasts into myofibroblast, accelerating cardiac remodeling and dysfunction. Cell therapies provide a novel direction for the enhancement of cardiac structure and function but remain poorly described in terms of the effect on resident cardiac fibroblasts. We have shown cortical bone derived stem cells (CBSCs) exhibit an ability to repair the heart after myocardial injury together with reduced scar formation. Nevertheless, whether CBSCs possess ability to modulate resident fibroblast response after myocardial injury remains untested.

**Objective:**

To determine the effect of secreted factors from CSBCs to attenuate myofibroblast formation in the heart after injury.

**Methods and Results:**

CBSCs were injected in mice after myocardial infarction which demonstrated reduced fibrosis as determined by Masson’s trichrome and Picro-Sirius red staining. In parallel, decreased expression of myofibroblast markers such as Acta2 was observed compared to PBS injected mice. To determine the effect of CBSCs on cardiac fibrosis, adult mouse cardiac fibroblasts were isolated from C57BL/6 mice, primed with CBSC pre-conditioned media for 12 h, and treated with 10ng TGF-β for 48 h to mimic cardiac injury. Decreased expression of Acta2, periostin and CTGF was observed in adult cardiac fibroblasts cultured in CBSC medium compared to control cells. Additionally, analysis of myofibroblast markers such as vimentin and pSMAD/SMAD was also decreased compared to control cells. To determine the mechanism, we looked for enriched miRNA in CBSCs that can mediate anti fibrotic response after injury. Results showed significantly increased expression of miR-18a in CBSCs. The upregulation of miR-18a was also validated in adult fibroblasts treated with CBSCs compared to control cells. Adult fibroblasts treated with mimic for miR-18a followed by TGF-β showed significant decrease in myofibroblast formation while miR-18a inhibitor completely inhibited the effect of CBSC medium.

**Conclusion:**

CBSCs reduce fibroblast to myofibroblast transition and differentiation in adult cardiac fibroblasts via miR-18a-5p. This finding reveals a new avenue for cell therapies to target myocardial scar modulation and provides a resolution for the cardiac repair response after injury in the adult myocardium.

## Introduction

Cardiac fibrosis is an outcome of most cardiac injuries promoting stiffness in the heart by excessive accumulation of the extracellular matrix (ECM). Deposition of increased ECM results in cardiac remodeling and scar formation leading to compromised cardiac contractility after injury (Berk et al., 2007; Kong et al., 2014). Since the myocardium is comprised of about 70% nonmyocytes with the majority including fibroblasts (Banerjee et al., 2007) it is therefore crucial to study pathways that can promote pro-reparative responses in fibroblasts to enhance repair after cardiac ischemic event. Cell-based therapies have recently provided a new direction for enhancement of cardiac structure and function after myocardial injury (Laflamme et al., 2005; Schachinger et al., 2006; Tang et al., 2010; Makkar et al., 2012; Hong S. J. et al., 2014; Karantalis et al., 2014). Transplanted stem cells can promote cardiac repair in the heart after injury by release of paracrine factors at the site of injury (Gnecchi et al., 2006, 2008; Duran et al., 2013; Hodgkinson et al., 2016). Studies have shown that stem cell derived paracrine factors promote cardioprotection (Sharp et al., 2017), myocyte cell cycle (Khan et al., 2015), and angiogenesis (Hatzistergos et al., 2010; Khan et al., 2015). Nevertheless, the effect of paracrine factors on the ability to modulate cardiac fibrosis and myofibroblast formation in the heart after injury remains poorly characterized.

Several studies recently have shown the salutary effects of multiple stem cell secretome on cardiac structure and function (Ibrahim et al., 2014; Gallina et al., 2015). Nevertheless, the main question is the selection of the optimal stem cell type for cardiac wound healing. We have recently shown that Cortical Bone-Derived Stem Cells (CBSCs) isolated and cultured from the cortical bone, preserved left ventricular (LV) volumes and ejection fraction in the hearts, as shown by echocardiogram in small (Duran et al., 2013) and large animal (Sharp et al., 2017) heart failure models. While these studies showed cardioprotective effects of CBSCs in vivo, the underlying mechanism of this effect is not yet known. One of the most significant observations during the animal studies with CBSCs were reduced fibrotic scar size after treatment with CBSCs. We hypothesized that CBSCs secretome possesses the ability to augment pro-reparative changes by suppressing the fibrotic response after injury which would explain the beneficial effects seen in the transplanted animals in previous studies. Since miRNAs represents one of the major components in the secretome that can ultimately affect cellular processes (Chiang et al., 2019), such as protection, survival and proliferation of cardiac cells (Borden et al., 2019), we looked for enriched miRNAs in CBSC the secretome with potential cardioprotective and anti-fibrotic effects. We observed expression of miR-18a-5p upregulated in animals after CBSC treatment.

In the article, we identify a novel role for CBSC secreted factors in modulating cardiac scar and fibrosis. Moreover, CBSCs attenuate myofibroblast formation via secretion of miR-18a-5p that represses CTGF mediated myofibroblast formation in the heart after injury. Our data suggests, CBSC secretome to possess potent scar remodeling and wound healing abilities providing a possible mechanism for the salutary effects of CBSC therapy for myocardial injury.

## Materials and Methods

### Primary Adult Cardiac Fibroblast Isolation and Treatment

Adult cardiac fibroblasts (ACFs) were isolated from the ventricular myocardium of 8–12-week-old adult C57/B6 mice. Briefly, ventricles were cut and washed with sterile 1X HBSS, and cut into fine pieces in digestion buffer (HBSS buffer, 100 U/mL of collagenase II, 2.5% trypsin) and transferred to a 50 mL sterile Falcon tube for digestion. The supernatant from digested tissue was then centrifuged, and pellets were combined and cultured in adult cardiac fibroblast media consisting of filtered DMEM/F12, 10%FBS, 100 U/mL pen/strep, 20 mM L-glutamine, and 0.1 mM 2-mercaptoethnanol, on collaged coated plates for 3 days until confluency. Cells were plated at 50,000 cells at passage 1 for 24 h in DMEM supplemented with 20% FBS on collagen coated plates and primed with CBSC pre-conditioned media (500,000 cells plated for 48 h) for 12 h and stimulated with 10 ng TGF-β for 48 h before harvesting.

### Cortical Bone Derived Stem Cell Culture and Characterization

Cortical bone derived stem cells are isolated from tibias and femurs of C57BL/6 mice and characterized as described previously (Duran et al., 2013; Mohsin et al., 2015). Briefly, tibias and femurs are flushed to get rid of all the bone marrow and then digested in collagenase at 37°C. The digested cells are washed and plated in CBSC media till colonies of CBSCs appear. CBSC media consists of DMEM-F12 mix supplemented with; 10% Embryonic Stem Cell Qualified FBS, 10 ml/L Pen/Strep/Glytamine (100× stock), 10 μL/L Leukemia Inhibitory Factor (1 mL/107 units), 10 mL/L ITS (100×), 40 ng/mL EGF, 20 ng/mL bFGF. The CBSCs are used from passage 12–16. The cells characterized for CBSC markers including CD44, CD105, CD106, Sca-1 and negative for CD45and then expanded for experiments.

### Quantitative Real-Time PCR and RT2 Profiler PCR Arrays

Fibroblasts and transplanted hearts were tested for expression of fibrotic genes by using RT2 profiler PCR arrays (Qiagen). Briefly, RNA was isolated from cells using the miRNeasy Kit (Qiagen) according to manufacturer’s protocol. Single-stranded cDNA was synthesized from all samples using the RT2 First Strand Kit (Qiagen) as described in the Qiagen protocol for RT2 profiler array sample preparation on an ABI stepOneplus system (Applied Biosystems). The primer sets used during the study is listed is in Table 1.

**TABLE 1:** List of primers.

Primer set	Forward	Reverse
Acta2	ACTCTCTTCCAGCCATCTTTC	GCTGTTATAGGTGGTTTCGTGG
Vimentin	TTCAAGAACACCCGCAGGAA	TTGGCAAAGCGGTCATTCAG
Collagen 1	AAGGAGACACTGGTGCCAAA	GGACCTTGAACTCCAGTAGC
Periostin	TGGAAGGGATGAAAGGCTGC	CCCAGCGTGCCATAAACATG
CTFG	CCCTCGCGGCTTACCGACTGG	CACAGG TCTTGGA ACAGGCGC
SMAD2	ACTAACTTCCCAGCAGGAAT	GTTGGTCACTTGTTTCTCC
18s	GGTCTTCGTCGGTAGGCATC	ACACCGACACGAGAGAGAGA
GAPDH	CATGGCCTTCCGTGTTCCTA	TACTTGGCAGGTTTCTCCAGG

### Western Blot

Western Blot analysis was performed as described previously (Borden et al., 2019). Briefly, sample concentrations were determined using the Bicinchoninic assay (BCA) according to manufacturer’s protocol and ran on a Mini-PROTEAN TGX Gels (Bio-rad). Primary antibodies against Acta2 (1:1000, rabbit polyclonal, Abcam, catalog ab5694), GAPDH (1:1000, mouse monoclonal, Millipore Sigma, catalog MAB374), CTGF (1:000, rabbit polyclonal, Abcam, catalog ab6992), P-SMAD2 (1:1000, rabbit polyclonal, Millipore Sigma, catalog ZRB04953), SMAD2 (1:1000, rabbit polyclonal, Abcam, catalog EP784Y), and Vimentin (1:1000, mouse monoclonal, Abcam, catalog ab8978) overnight at 4°C, and incubated with the appropriately conjugated light-sensitive IRDye secondary antibodies (1:5000, LiCOR) for 1 h at room temperature, and visualized.

### Immunocytochemistry

Cells were prepared for immunocytochemistry as described earlier (Mohsin et al., 2015). Briefly, cells were fixed on tissue-chamber slides with 4% paraformaldehyde and washed with PBS and 0.1% Triton X-100 and blocked for 30 min with 0.5% Horse serum. Cells were then incubated with rabbit anti-Acta2 (1:100, rabbit polyclonal, Abcam, catalog ab5694) and DAPI (0.1 μg/mL in PBS) (EMD Millipore, catalog 268298-10MG) (light-sensitive) for 10 min at room temperature and mounted on slides for imaging.

### Induction of Acute MI and Fibrosis Detection

Animals (C57BL/6 8–12 weeks old male mice) *n* = 30 were divided into two groups PBS and CBSC treated). All surgical procedures and animal care protocols were approved by the Temple University Animal Care and Use Committee. Animals underwent myocardial infarction procedure by permanent ligation of the left anterior descending artery (LAD) as described previously (Duran et al., 2013). 100,000 CBSCs were injected in the border zone area at the time of infarction. The animals were sacrificed 2 weeks after MI for Masson Trichome and Picro-Sirius red staining following manufacturer’s protocol. Briefly, formalin-fixed heart tissues were routinely processed, embedded in paraffin, and sectioned for histochemistry. Masson’s Trichrome staining was performed with Trichrome stain kit (HT 15-1 KT, Sigma-Aldrich) and Weigert’s Iron Hematoxylin (HT1079-1SET Sigma Aldrich). Picro-Sirius red staining was performed with the kit (ab150681, Abcam) briefly following the steps of section deparaffinization, incubation with staining solution and dehydration. Pictures were taken with light microscopy. Fibrosis and non-fibrosis areas were calculated with ‘color threshold’ tool from ImageJ software (version 1.49v; National Institutes of Health).

### microRNA Treatments

Adult cardiac fibroblasts were transiently transfected with 50 nM miR-18a-5p mimic (Thermo Fisher Scientific, catalog 4464066), miR-18a-5p inhibitor (Thermo Fisher Scientific, catalog MH12973), or negative control (Thermo Scientific, catalog 4464058) using Invitrogen Lipofectamine 3000 (Thermo Fisher Scientific, catalog L3000015) in serum free Gibco Opti-MEM media (Thermo Fisher Scientific, catalog 31985062) according to the manufacturer’s recommendations. After 24 h post transfections, cells were used for protein and RNA analysis or stimulated with 10ng TGF-β for 48 h before harvesting.

### Luciferase Assay

Adult cardiac fibroblast (30,000 cells on 12 well plate) were plated and the following day transiently transfected with Lipofectamine 3000 (Thermo Fisher Scientific, catalog L3000015) and CTGF miRNA (GeneCopoeia) in Gibco Opti-MEM media (Thermo Fisher Scientific). Cells were treated with 50 nM miR-18a-5p mimic or inhibitor as described previously. The luciferase assay was performed using the GeneCopoeia Luc-Pair Duo-Luciferase Assay Kit 2.0 (GeneCopoeia). Cells were prepared and washed according to the manufacturer’s recommendations. The ratio of luminescence was measured on luminometer.

### Animal Housing and Husbandry Care

All animals are housed within our AAALAC accredited animal facility. The facility is staffed with veterinary technicians and husbandry staff. The mice are housed 4–5 mice/cage. All animals are purchased through Jackson Laboratories. Each suite contains housing rooms and small animal surgical wards to perform necessary testing in a centralized, controlled environment to minimize animal stress. The animals are provided with comprehensive preventative medicine and veterinary care programs including daily observation of animals (weekends and holidays) by husbandry staffing, veterinary technicians, and on-site veterinarians. The primary method of euthanasia that will be used is CO_2_ inhalation. Euthanasia procedures will be conducted in accordance to the recommendations published by the Panel of Euthanasia of the American Veterinary Medical Association.

### Statistical Analysis

Data are represented as mean ± SD. Two-sided testing was used for all statistical tests. *P* values of ≤0.05 was used to determine significance for all statistical tests. Comparisons for data with a single measurement were performed using the unpaired *t*-test. Analysis was performed by One-or two way ANOVA (analysis of variance), followed by Tukey’s multiple comparison test using the GraphPad Prism software (GraphPad Inc., La Jolla, CA, United States).

## Results

### CBSCs Treatment Restrict Fibrotic Scar After MI

**FIGURE 1:**
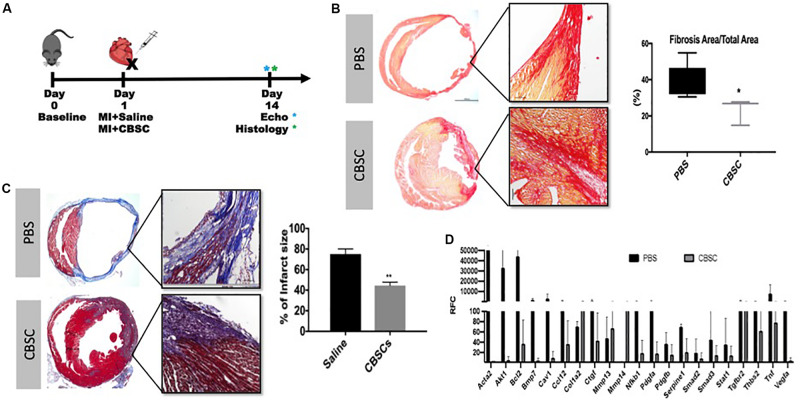
CBSCs treatment restrict fibrotic scar after MI. **(A)** Outline of experimental design using an adult mouse heart given a myocardial infarction (MI) and treated with CBSC or Saline injection with measurements taken 3, 7, and 14 days after injection. **(B)** Fibrosis was decreased in hearts injected with CBSCs compared to PBS using Picrosirius Red stain for fibrosis and quantified based on the amount of fibrosis relative to the total area 14 days after injection, *n* = 5, *P < 0.05. **(C)** A decrease in percent of infarct size in heart injected with CBSCs compared to PBS hearts using Masson’s trichrome staining and quantified based on the percentage of infarct size 14 days after injection, *n* = 5, **P < 0.01. **(D)** A fibrotic screen of a MI heart treated with PBS or CBSCs showing lower myofibroblast marker expression in CBSC treated hearts compared to PBS treatment. Black bars indicating the control PBS or Saline treatment with adult cardiac fibroblasts, gray bars indicating CBSC treatment with adult cardiac fibroblasts.

Animals treated with CBSCs after myocardial infarction (experimental design illustrated in Figure 1A) showed reduction in scar size measured by Masson’s Trichrome staining and Picro-Sirius red staining after treatment (Figures 1B,C). The quantitative analysis showed 1.6-fold decrease in fibrotic scar versus saline treated animals (*p* < 0.05) by the histological analysis. Concurrent with reduced scar size, we showed expression of fibroblast and myofibroblast markers including Acta2, CTGF, SMAD2 and SMAD 3 are down regulated compared to Saline treated group (Figure 1D and Supplementary Figure 1). These findings conclude that CBSCs treatment helps with restriction of fibrotic scars explaining the functional benefits observed in the earlier studies.

### CBSC Secretome Reduces Myofibroblast Markers in Adult Cardiac Fibroblasts

**FIGURE 2:**
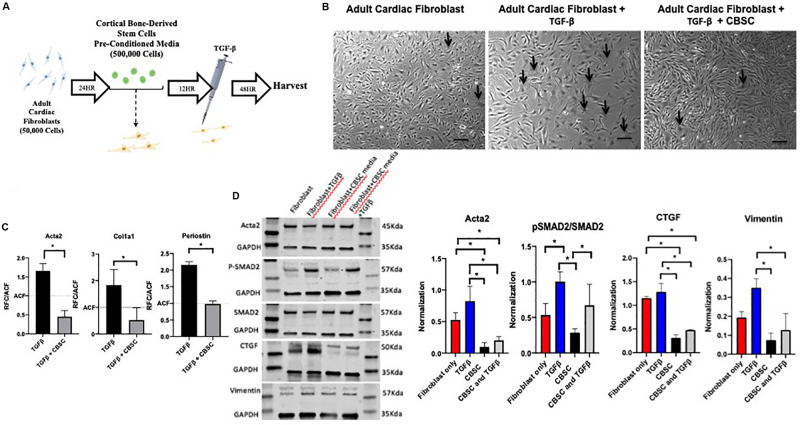
CBSC secretome reduces myofibroblast markers in adult cardiac fibroblasts. **(A)** Outline of experimental design showing adult cardiac fibroblasts isolation **(B)** Representative image of untreated fibroblasts that have a (spindle-like shaped), fibroblast treated with TGFβ (flattened and broad) indicating myofibroblast transition. The fibroblast reverts their morphology back to untreated fibroblasts after CBSC treatment scale bar represents 20 μM. **(C)** Decreased myofibroblast marker expression is observed in fibroblast treated with CBSCs and TGFβ measured by quantitative reverse transcription polymerase chain reaction analysis, *n* = 3, *P < 0.05. **(D)** Decreased myofibroblast and TGFβ pathway maker expression are observed in fibroblasts treated with CBSCs and CBSCs and TGFβ using Western blot analysis, normalized to GAPDH, *P < 0.05.

Adult cardiac fibroblasts were cultured as demonstrated in Supplementary Figure 1. The purity of our adult cardiac fibroblast cultures and their transition to myofibroblasts was confirmed by staining with Acta2 after TGF-β stimulation. As anticipated stress fibers were formed and stained with Acta2 indicating myofibroblast transition (Supplementary Figure 3). Concurrently, markers including Acta2, Periostin and Col1a was significantly increased after TGF-β treatment as measured by RT-PCR analysis (Supplementary Figure 3). We wanted to confirm whether CBSCs secretome is inhibiting fibroblasts to myofibroblasts transition, therefore, we treated adult cardiac fibroblasts with CBSC pre-conditioned media for 12 h, followed by stimulation with 10 ng TGF-β for 48 h (Figure 2A). Untreated fibroblasts have a spindle-like shape cells as confirmed by a light microscopy which is significantly changed to flattened broad cells after introduction of TGF-β stimulation. However, we see a reversal of morphology of stimulated fibroblasts after pretreatment with CBSCs (Figure 2B). These findings were further confirmed by analyzing the stimulated and CBSC treated adult cardiac fibroblast for the myofibroblast markers using RT PCR analysis. There was a significant reduction in the gene expression of myofibroblast markers including Acta2, Cola1a1 and Periostin (*p* < 0.05) (Figure 2C). Simultaneously, there was a decrease protein expression of Acta2 (3-fold with *p* < 0.05) and CTGF (3.6-fold with *p* < 0.05) in adult cardiac fibroblast versus CBSCs treated fibroblasts after TGF-β stimulation. Additionally, expression of the ratio between PSMAD2 to SMAD2 (1.66-fold) and Vimentin (3-fold) also showed significant decrease after CBSC treatment alone and after TGF-β stimulation in adult cardiac fibroblasts (Figure 2D), indicating a decrease in TGF-β signaling pathway with CBSC treatment. These findings clearly demonstrate the role of CBSC secretome in reducing fibrotic genes/proteins, suggesting the CBSC secretome’s role in suppressing fibrogenic response after injury.

### miR-18a-5p Prevents Adult Cardiac Fibroblast Differentiation

**FIGURE 3:**
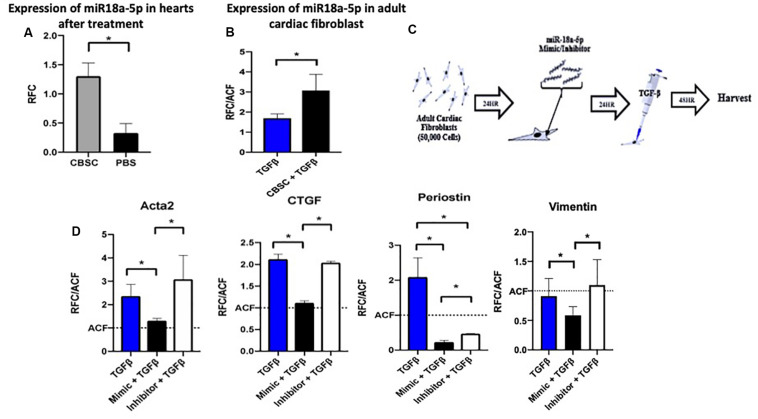
The miR-18a-5p prevents adult cardiac fibroblast differentiation and TGF-β stimulation. **(A)** The increased expression of miR-18-5p in adult hearts after treatment with CBSCs compared to PBS treatment using quantitative reverse transcription polymerase chain reaction analysis, *n* = 4, *P < 0.05. **(B)** The increased expression of miR-18-5p in adult cardiac fibroblast treated with CBSC and TGFβ compared to TGFβ alone using quantitative reverse transcription polymerase chain reaction analysis, *n* = 3, *P < 0.05. **(C)** Outline of experimental design using adult cardiac fibroblasts treated with the miR-18-5p mimic or inhibitor for 24 h. **(D)** Decrease in myofibroblast marker expression in fibroblast treated with the mimic of miR-18-5p with TGFβ compared to TGFβ alone and/or the inhibitor of miR-18-5p and TGFβ using quantitative reverse transcription polymerase chain reaction analysis, *n* = 3, *P < 0.05 All qRT-PCR data is represented as RFC (relative fold change).

After confirming that CBSC secretome significantly reduce the fibrotic response after injury in vivo and in vitro using adult cardiac fibroblast, we wanted to look for the potential mechanism. Since stem cell secretome represents a large fraction of miR’s we screened the hearts treated with CBSCs for miRNAs with roles in the fibrotic response. Expression of mir-18a-5p was 3.25-fold higher in hearts treated CBSC versus saline treated hearts after MI (Figure 3A). We additionally confirmed the increased expression of miR-18a-5p in fibroblasts treated with CBSC pre-conditioned media after TGF-β stimulation (Figure 3B). When we screened for highly expressed miRNA’s in cultured CBSCs, that can mediate an anti-fibrotic response we identified miR-18a-5p (Supplementary Figure 4A). Additionally, we also confirmed expression of miR-18a-5p physiological levels in mouse hearts, the expression goes down 6-fold from 2 days versus 2 weeks after birth (Supplementary Figure 4B). Similarly, in an isolated cardiac fibroblast the expression of miR18a-6p was significantly reduced (2-fold) in fibroblasts isolated from neonatal versus adult heart (Supplementary Figure 4C). To further validate the finding, the adult cardiac fibroblasts were dosed with miR-18a-5p mimic or inhibitor overnight before treated the cells with TGF-β for 48 h (Figure 3C). The effect of the mimic and inhibitor with TGF-β was measured using RT-PCR (Figure 3D). TGF-β stimulated fibroblast with the miR-18a-5p mimic had decreased expression levels of myofibroblast markers including Acta 2, CTGF, Periostin and Vimentin (Figure 3D) versus miR-18a-5p inhibitor treatment indicating a more differentiated state. This helped elucidate the importance of miR-18a-5p in preventing adult cardiac fibroblast differentiation into myofibroblasts.

### miR-18a-5p Prevents Fibrosis by Targeting CTGF After Injury

**FIGURE 4:**
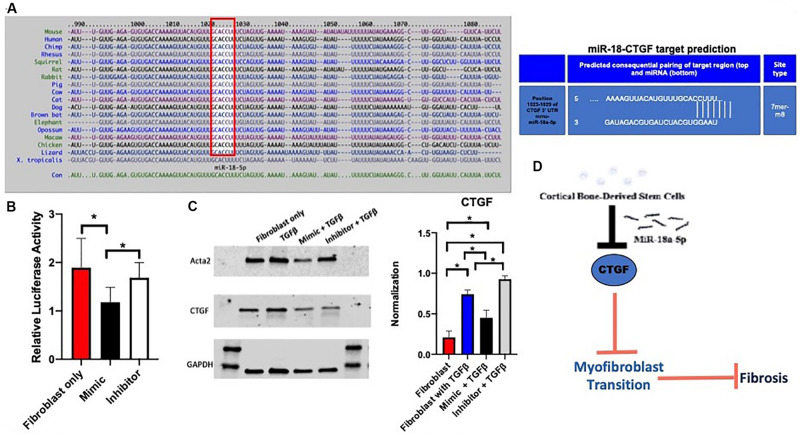
The miR-18a-5p prevents fibrosis in the adult heart. **(A)** A target scan search of the miR-18-5p sequence indicating conserved sequence across species and a miR-18-5p target predication position for pairing of the target region of the myofibroblast marker, CTGF. **(B)** Decrease in relative luciferase activity of CTGF with the treatment of the mimic of miR-18-5p using a Luc-Pair Duo-Luciferase assay, *n* = 6. **(C)** A decrease in myofibroblast marker expression of Acta2 and CTGF respectively with the treatment of the mimic of miR-18-5p with TGFβ using western blot analysis, normalized to GAPDH, *n* = 6, *P < 0.05. **(D)** The mechanism of action of CBSCs and the miR-18-5p inhibiting CTGF, preventing the myofibroblast transition and fibrosis.

miRNAs act through transcriptional repression and targeting of mRNA 3′-UTR, so a miRNA target prediction search was conducted using TargetScan 7.2 that identified CTGF as a potential target with only 1 putative 7mer site within CTGF-3′-UTR (Figure 4A) among the fibrotic genes altered. To confirm whether miR-18a targets CTGF-3′-UTR, a reporter assay was performed using 3′-UTR of CTGF that drives luciferase expression. Adult cardiac fibroblasts were transfected with mouse CTGF 3′-UTR luciferase reporter plasmid together with control plasmid and treated with miR-18a mimic or inhibitor. Treatment with miR-18a mimic reduced luciferase activity validating miR-18 targeting of CTGF (*p* < 0.05) (Figure 4B). After confirming the CTGF as a target for miR-18a, western blot analysis was performed which showed a significant decrease in myofibroblast markers including Acta2 and CTGF (*p* < 0.05) after treatment of TGF-β and miR-18a-5p mimic, while there was an increase with the miR18a inhibitor treatment (Figure 4C). Therefore, we conclude that CBSC secretome release miR18a-5p which blocks CTGF and inhibits the fibrogenic response (Figure 4D).

## Discussion

Our findings here identify a novel role for cortical bone derived stem cells (CBSCs) secreted factors in modulating cardiac fibrosis in the heart after myocardial injury. In particular, CBSCs secreted factors attenuate myofibroblast formation via release of miR-18-5p which represses connective tissue growth factor (CTGF) leading to concurrent reduction in signaling pathways associated with myofibroblast formation.

Cardiac fibrosis is one of the most predominant outcomes of heart failure during ischemia (Sutton and Sharpe, 2000). Cardiomyocyte death triggers a massive inflammatory and fibrogenic response that initially is largely an adaptive response leading to scar formation designed to prevent cardiac rupture (van den Borne et al., 2010; Shinde and Frangogiannis, 2014). However, cardiomyocyte death leads to adverse cardiac remodeling and fibrosis continues to progress and transition into replacement fibrosis, which is associated with further cardiomyocyte loss leading to ventricular dilation and heart failure (Janicki and Brower, 2002; Berk et al., 2007; Kong et al., 2014). During this later phase, tissue-resident cardiac fibroblasts (CFs) become activated into stellate-shaped cells called myofibroblasts (MFs), characterized by increased expression of α-smooth muscle actin (Acta2), Collagen I, and Periostin (van den Borne et al., 2010). Current available therapies and treatments improve cardiac function and slow down the progression of the disease but are unable to reverse cardiac scar as a consequence of cardiomyocyte death. In the past decade, cell-based therapies have offered a new way to address cardiac repair and regeneration after injury (Bolli et al., 2011; Kanazawa et al., 2015). Several adult stem cells have demonstrated that donated cells in the heart improve cardiac structure and function after myocardial injury (Karantalis et al., 2014; Kanazawa et al., 2015). Nevertheless, adoptively transferred stem cells are lost in the heart early yet the effects persist for up to a year indicating additional mechanisms at play (Hong K. U. et al., 2014). Moreover, salutary effects of cell therapy are linked to the secretion of paracrine factors at the site of injury that mimic similar responses as the parent cell in enhancing cardiac function (Mayourian et al., 2018). Stem cell derived paracrine factors have been shown to exert cardioprotective effects together with promoting cardiomyocyte proliferation and angiogenesis. Interestingly, the effect of cell-based therapies to modulate cardiac wound healing and cardiac fibroblast response have been largely overlooked. We have previously employed CBSCs isolated from the bone cortex for myocardial repair and the data shows CBSC therapy enhanced cardiac structure and function (Duran et al., 2013; Sharp et al., 2017). Importantly, CBSCs transplanted animals showed significantly decreased infarct size and scar suggesting a possible role for CBSCs in modulation of cardiac wound healing. In this context, we show here that CBSC secretome releases factors that target adult cardiac fibroblasts preventing transformation into myofibroblasts and thereby promoting cardiac function.

Our results show that CBSC secrete a number of cardioprotective factors including miR-18a-5p. miR-18a5p belongs to the miR-17-92 cluster with seed sequence AAGGUG and participates in a number of physiological processes regulating proliferation, migration and differentiation of various cell types (Hayashita et al., 2005; Mendell, 2008; Gu et al., 2017). In cardiac biology, miR-18a-5p expression declines in patients with heart failure and is considered to be a biomarker for cardiovascular diseases (Ovchinnikova et al., 2016). Nevertheless, there is not much evidence of miR-18a-5p in cardiac fibroblasts or cardiac fibrosis after myocardial injury. Our results show that adult cardiac fibroblasts treated with CBSCs medium increase miR-18a-5p expression suggesting the ability of CBSCs to transfer miR-18a-5p to adult fibroblasts. Moreover, miR-18a-5p expression declines in the heart after myocardial infarction together with reduction of cardiac fibrosis and decreased fibroblast-myofibroblast transition in response to TGF-β treatment. During cardiac injury myofibroblast transformations rely heavily on the transforming growth factor (TGF-β) signaling pathway (Khalil et al., 2017). Fibroblast-myofibroblast transition (FMT) pathways have been shown to be controlled by TGF-β signaling, that upregulates mesenchymal markers such as Acta2 and fibronectin, markers associated with the increase in differentiation expression (Kim, 2018). We observe that CBSCs reduce the fibroblast-myofibroblast transition in adult cardiac fibroblasts by overexpressing miR-18a-5p. We found that CBSCs counteract increased differentiation created by TGF-β stimulation, reducing genes overexpressed by the extracellular matrix and promoting wound healing processes. Mechanistically, our results showed that miR-18a-5p targets connective tissue growth factor (CTGF) in the adult cardiac fibroblasts. Several studies recently have implicated a central role of CTGF in regulating tissue remodeling and fibrosis (Sakai et al., 2017; Dorn et al., 2018; Ramazani et al., 2018). CTGF expression is induced by many cytokines and conditions associated with pathophysiology of different organs (Holbourn et al., 2008; Jaffa et al., 2008; Chen and Lau, 2009). Its presence induces formation of myofibroblasts by differentiation of other cell types, activates myofibroblasts and stimulates their deposition and remodeling of ECM protein. In the heart, CTGF expression is induced after myocardial injury and its blockade is associated with reduced cardiac fibrosis (Chatzifrangkeskou et al., 2016; Tan et al., 2019). In accordance, our results suggest that CBSC mediated enhancement of the cardiac structure and attenuation of cardiac fibrosis is mediated via secretion of miR-18a-5p that in turn blocks CTGF expression in adult cardiac fibroblasts leading to reduced myofibroblast formation and ECM remodeling.

In conclusion, we report here a novel role of CBSC secretome in modulating cardiac fibrosis via release of miR-18a-5p that targets adult cardiac fibroblasts attenuating their ability to form myofibroblasts, thereby enhancing cardiac structure and function.

## Data Availability Statement

All datasets generated for this study are included in the article/Supplementary Material.

## Ethics Statement

The animal study was reviewed and approved by Temple University Animal Care and Use Committee.

## Author Contributions

LK and LM contributed equally to the design, data, and writing of the study. YY helped with data in a section. FN helped with formatting and data. RH and TO helped with data analysis. MK and SM helped with design and sections of the manuscript. All authors contributed to the manuscript revision, read and approved the submitted version.

## Conflict of Interest

Dr. Mohsin is a named inventor on intellectual property fillings that are related to cells used in the manuscript. The remaining authors declare that the research was conducted in the absence of any commercial or financial relationships that could be construed as a potential conflict of interest.

## Nomenclature

ACF: adult cardiac fibroblasts
Acta2: alpha smooth muscle actin
CBSC: cortical bone stem cells
CF: cardiac fibroblast
Col1a1: collagen type 1 alpha 1
CTGF: connective tissue growth factor
ECM: extracellular matrix
FMT: fibroblast-myofibroblast transition
HBSS: Hank’s balanced salt solution
LAD: left anterior descending artery
LV: left ventricular
MF: myofibroblast
MI: myocardial infarction
PBS: phosphate buffered saline
TGF: transforming growth factor beta.
