# Supplementary material for: Cortical Bone Derived Stem Cells Modulate Cardiac Fibroblast Response via miR-18a in the Heart After Injury
Source: Front Cell Dev Biol. 2020 Jun 23;8:494. doi: 10.3389/fcell.2020.00494 (PMC7324629; doi:10.3389/fcell.2020.00494)
Supplement: Supplementary file 1 [file Data_Sheet_1.PDF]

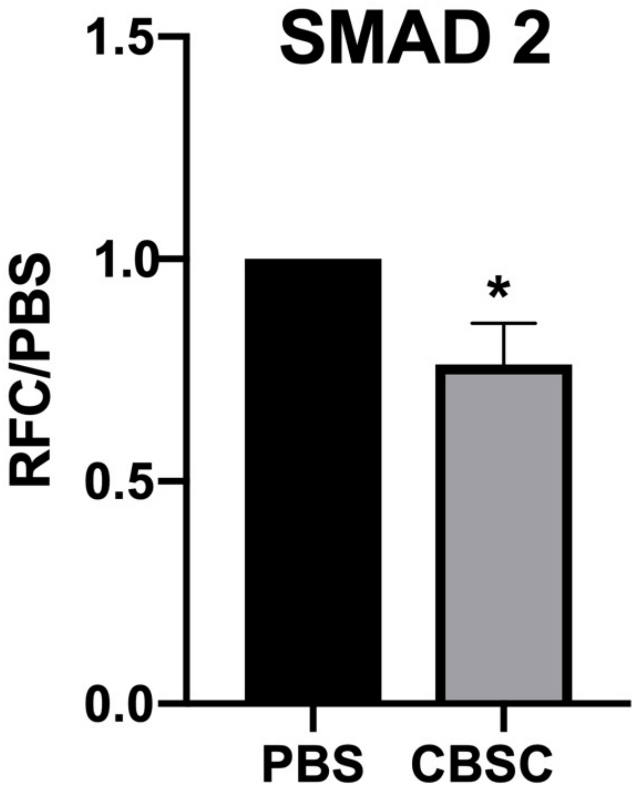

Supplemental Figure 1: Expression of SMAD 2 after PBS versus CBSC treatment after Cardiac injury.

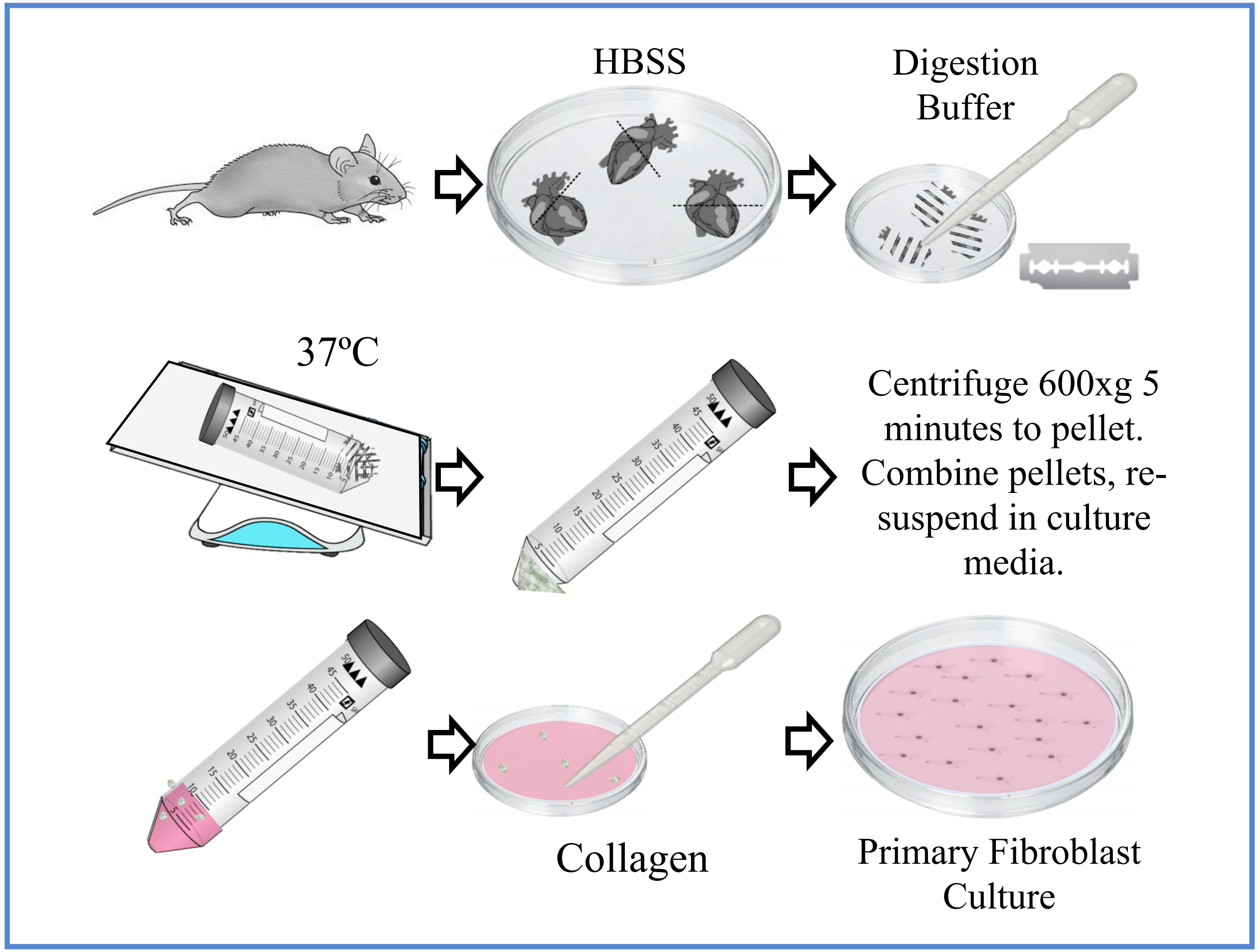

Supplemental Figure 2: Illustration for adult cardiac fibroblasts isolation: Heart ventricles are isolated from 8-12 week adult mice, digested in collagenase II and trypsinized at 37ºC, pelleted, and re-suspended in DMEM with high glucose and sodium pyruvate, supplemented with 20% FBS, and plated on collagen-coated petri dish for 90 minutes.

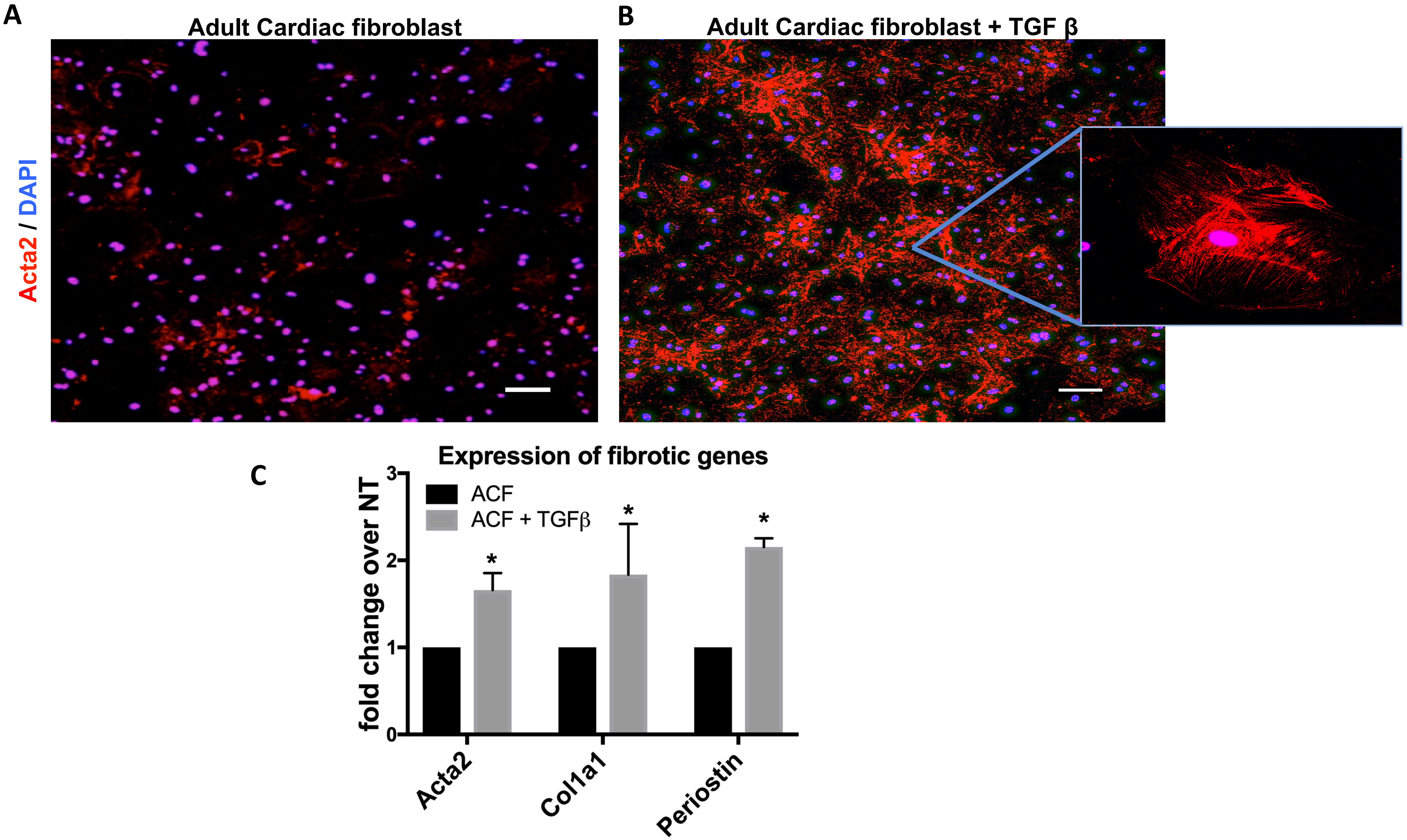

Supplemental Figure 3: Characterization of myofibroblast: A; Adult cardiac fibroblasts B; Adult cardiac fibroblasts stimulated with TGFb, Acta 2 in red and DAPI in blue C; Expression of fibrotic genes including Acta2, Col1a1 and periostin is upregulated after TGFb treatment measured by RT-PCR analysis.

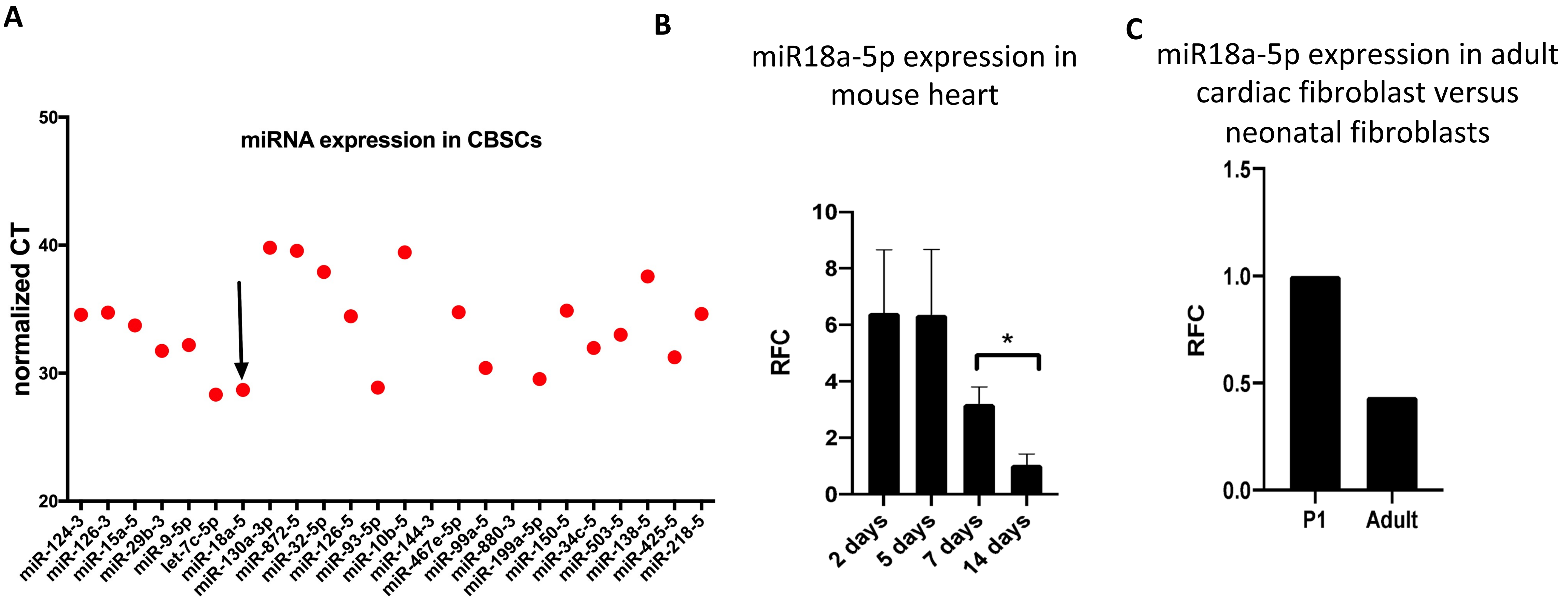

Supplemental Figure 4: The miR-18a-5p expression in CBSCs, mouse hearts and isolated fibroblasts. A; Expression of different miRNA in CBSCs, values are expressed as normalized CT therefore the smaller value correlates to high expression of miRNA. Expression of miR18a-5p is indicated by an arrow. B; The decrease in miR-18a-5p expression in adult hearts over 14 days using quantitative reverse transcription polymerase chain reaction analysis, *$P<0.05$. C, The decrease in miR-18a-5p expression in adult cardiac fibroblasts compared to neonatal fibroblasts using quantitative reverse transcription polymerase chain reaction analysis**.**
